# The Effect of Liraglutide on Axon Regeneration and Functional Recovery after Peripheral Nerve Lesion

**DOI:** 10.3390/cimb46010021

**Published:** 2024-01-02

**Authors:** Mehmet Burak Yalçın, Ejder Saylav Bora, Oytun Erbaş

**Affiliations:** 1 Department of Orthopedics and Traumatology, Bahcelievler Memorial Hospital, Istanbul 34180, Türkiye; mehmetburakyalcin@gmail.com; 2 Department of Emergency Medicine, Izmir Atatürk Research and Training Hospital, Izmir 35360, Türkiye; 3 Department of Physiology, Demiroğlu Bilim University, Istanbul 34180, Türkiye; oytunerbas2012@gmail.com

**Keywords:** peripheral nerve injuries, GLP-1, GDF-11, galactin-3

## Abstract

Peripheral nerve injuries inflict severe consequences, necessitating innovative therapeutic strategies. This study investigates the potential of liraglutide, a glucagon-like peptide-1 receptor agonist, in mitigating the consequences of peripheral nerve injury. The existing treatment methods for such injuries underscore the importance of ongoing translational research efforts. Thirty adult Wistar rats underwent sciatic nerve dissection and repair surgery. The nerves were surgically transected using micro scissors at a precise location located 1.5 cm proximal to the trifurcation site. The study included a control group and two experimental groups, one treated with saline (placebo group) and the other with liraglutide (experimental group) for 12 weeks. Motor function, electromyography (EMG), and biochemical and histopathological analyses were performed after 12 weeks of treatment. Electrophysiological assessments revealed that liraglutide improved the compound muscle action potential (CMAP) amplitude and motor function compared to the saline-treated group. Histological and immunohistochemical analyses demonstrated increased NGF expression, total axon number, and diameter and reduced fibrosis in the liraglutide group. Biochemical analyses illustrated liraglutide’s antioxidative properties, evidenced by reduced malondialdehyde (MDA) levels. Galectin-3 levels were suppressed and GDF-11 levels were modulated by liraglutide, indicating anti-inflammatory and anti-apoptotic effects. Liraglutide is a promising therapeutic intervention for peripheral nerve injuries, promoting functional recovery and histopathological improvement. Its multifaceted positive impact, beyond glycemic control, suggests constructive effects on the acute and chronic inflammatory processes associated with peripheral neuropathy. These findings warrant further research to elucidate molecular mechanisms and facilitate clinical translation. The study contributes valuable insights to the growing understanding of GLP-1 receptor agonists’ neuroprotective properties in the context of peripheral nerve injuries.

## 1. Introduction

Peripheral nerve injuries (PNI) significantly impact individuals, as evidenced by an estimated annual incidence of approximately 2 PNIs per 10,000 [1,2,3]. These injuries are known for their severe and life-altering consequences. The adverse health outcomes and unintended detrimental consequences, encompassing both physical and psychological aspects, resulting from the existing treatment methods underline the importance of ongoing translational research endeavors [2].

Injuries to the peripheral nerves can be caused by various traumatic experiences, resulting in the loss of one or more functions, including sensory, motor, and autonomic capabilities. Injuries to the nerves caused by trauma interfere with the normal conduction of nerve impulses [4]. Studies using EMG equipment have shown a slowdown in the nerve conduction process and the presence of fibrillation potentials and giant motor unit potentials [5,6].

The nerve regeneration process after nerve injury is a complex cellular phenomenon encompassing various elements such as inflammation, neurotrophic factors, neurotransmitters, adhesion, axon formation, growth cone development, and neuronal survival [7]. PNI initiates a cascade of immunoregulatory responses within the cellular microenvironment, leading to alterations in immune cells and associated immunoregulatory factors [8].

Liraglutide is a pharmacological agent that belongs to the class of long-acting synthetic analogs of glucagon-like peptide-1 (GLP-1), which has been utilized in managing type 2 diabetes [9]. GLP-1, an endogenous hormone, falls under the category of incretins and is released by enteroendocrine cells situated in the gastrointestinal tract following food consumption. The compound exhibits insulinotropic characteristics through activating the GLP-1 receptor (GLP-1R), a G-protein coupled receptor located on the beta cells of the pancreas. The endogenous hormone GLP-1 is subject to swift metabolism mediated by the enzyme dipeptidyl peptidase (DPP)-4, producing an inert peptide [10]. In contrast, GLP-1 receptor agonists (GLP-1RAs) possess a notably extended half-life in the circulatory system compared to GLP-1. The main factor contributing to their capacity to withstand enzymatic degradation, specifically facilitated by DPP-4, can be ascribed to them. GLP-1 receptors, called GLP-1Rs, are found in the pancreatic region and extrapancreatic tissues, including the central nervous system (CNS) and peripheral nervous system (PNS).

Moreover, a significant proportion of the agonists can cross the blood–brain barrier. Recently, a growing scholarly focus has been placed on the neurotrophic and neuroprotective properties exhibited by GLP-1 receptor agonists [11]. Several recent studies have presented findings indicating that glucagon-like peptide-1 receptor agonists might possess advantageous properties for neurodegenerative disorders [12,13,14,15].

Liraglutide has demonstrated notable efficacy in ameliorating the symptoms and neuropathological manifestations of Alzheimer’s disease (AD) and Parkinson’s disease (PD) in animal models. In addition, the above-mentioned neuropathological diseases have shown promising results in clinical trials, as evidenced in previous studies [16,17]. The documented literature also provides evidence of the neuroprotective effects of liraglutide on the peripheral nerve. The substances mentioned above have been observed to possess properties that promote the regeneration of axons and improve the recovery of normal function following injury to the sciatic nerve [16,17].

Growth differentiation factor 11 (GDF11) is a member of the transforming growth factor-β family identified relatively recently. The significant role of GDF11 in physiology, specifically embryogenesis, has been established as it plays a crucial part in bone formation. GDF11 has been characterized as a rejuvenating and anti-aging molecule with the potential to restore various functions. In addition to its role in embryogenesis, GDF11 is involved in the mechanisms underlying inflammation and carcinogenesis. Experimental studies have demonstrated that GDF11 exhibits anti-inflammatory properties in colitis, psoriasis, and arthritis [18]. The liver fibrosis and renal injury data suggest that GDF11 exhibits pro-inflammatory properties [19].

Galectins, a group of animal lectins that bind to beta-galactosides, play a significant part in various physiological and pathological processes [20]. Our research has primarily centered on the examination of two prominent galectins, namely galectin-1 (GAL-1) and galectin-3 (GAL-3), in their capacity as regulators of axonal degeneration and regeneration within the PNS [21]. It is predominantly released during monocyte-to-macrophage differentiation [22]. The phenomenon of PNS regeneration following injury is widely recognized. The plasticity of Schwann cells contributes to the successful regeneration and reinnervation in the PNS [23]. Following an injury, Schwann cells exhibit an upregulation of galectin-3, a lectin that binds explicitly to galactose. This upregulation has been linked to the phagocytosis of myelin during Wallerian degeneration [24]. 

This study uses electrophysiological, biochemical, and histopathological methods to show liraglutide’s protective and regenerative effect in a peripheral nerve injury experimental model.

## 2. Materials and Methods

### 2.1. Animal

The study used 30 adult male Wistar rats weighing 200–210 g. Under standardized circumstances, the animals were kept in enclosures, including a 12 h light–dark cycle at 22 ± 2 °C, relative humidity at 51 ± 3%, and airflow at 7 m·s^−1^. The subjects ate a pellet diet and received unlimited tap water throughout the research. This study uses Merck KGaA, (Darmstadt, Germany) chemicals unless otherwise noted.

**Figure 1:**
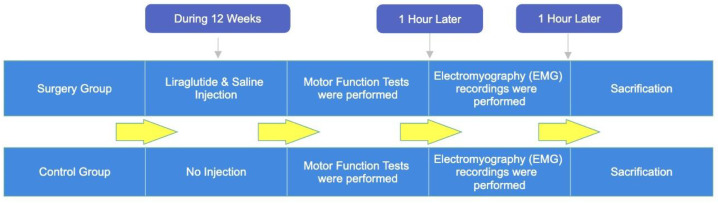
Time flow diagram of the study.

Twenty rats were selected for sciatic nerve dissection and repair surgery. Ten rats were chosen as the control group (n = 10) and received no surgery or medication. Ten rats who had surgery were randomly assigned to a placebo group and given saline. The rats received 1 mL/kg intraperitoneal 0.9% sodium chloride. In the surgery and liraglutide (experimental) group (n = 10), rats received 1.8 mg/kg/day [25] intraperitoneally. The Novo Nordisk brand Victoza, 6.0 mg/mL, was used. The medication was given for 12 weeks. Motor function was assessed after 12 weeks (Figure 1). The motor function assessment was followed by electromyography (EMG) in the nerve conduction study (NCS) context. The nerve conduction study (NCS) is a neurophysiological technique using electrodes (usually superficial) to study the conduction of action potentials in peripheral nerves.

Following the administration of anesthesia, all animals were euthanized using cervical dislocation. The anesthesia consisted of 100 mg/kg Ketasol (Richterpharma AG, Wels, Austria) and 50 mg/kg Rompun (Bayer, Leverkusen, Germany). Following the heart puncture procedure, blood samples were subjected to biochemical analysis. Subsequently, sciatic nerve samples were collected to conduct immunohistochemical and biochemical analyses.

### 2.2. Surgical Procedure 

The rats were subjected to general anesthesia using an intraperitoneal injection of a combination of ketamine (75 mg/kg) and xylazine (10 g/kg). Following that, the rats were carefully placed in a supine position on the surgical table. The sciatic nerves’ right and left sciatic nerve exposure was performed using an aseptic technique, commencing 1 cm distal to the sciatic notch and continuing 1 cm distal to the nerve’s trifurcation. The nerve segments, approximately 3–3.5 cm long and proximal to the trifurcation, were carefully dissected to isolate the sciatic nerve from the surrounding soft tissue. Following that, the nerves were surgically transected using micro scissors at a precise location located 1.5 cm proximal to the trifurcation site. This indicates the point of origin of the tibial nerve, common peroneal nerve, and caudal sural cutaneous nerve. The surgeon employed three epineural sutures (Ethilon^®^ 9-0, Ethicon, Raritan, NJ, USA) to reestablish the nerves’ connection. Following the procedure, the incision was closed using a 3-0 Vicryl^®^ material. Subsequently, the rats were provided with a period of recovery. After the rats regained consciousness from anesthesia, they were returned to their enclosures and provided unrestricted food and water resources.

### 2.3. Electrophysiological Recordings 

**Figure 2:**
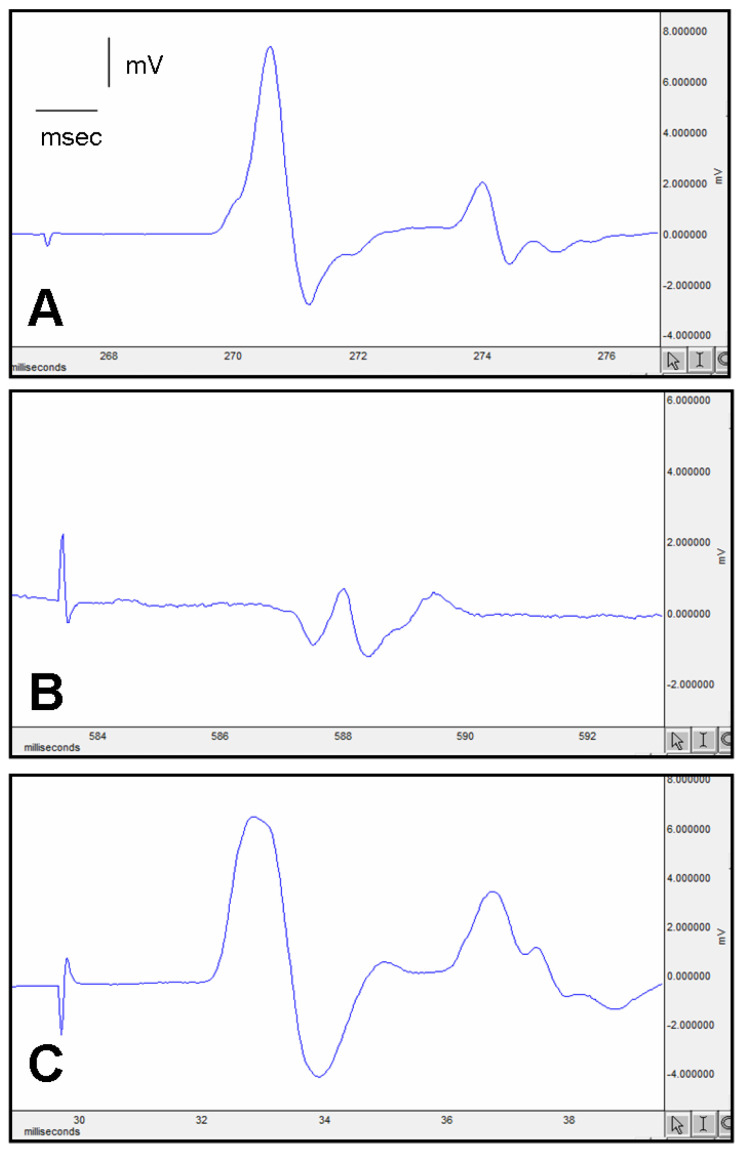
Demonstrative electromyography results of (**A**)—control group EMG, (**B**)—surgery and saline group EMG, and (**C**)—surgery and liraglutide group EMG.

The rats were subjected to anesthesia through the administration of a combination of ketamine hydrochloride (Alfamine, Alfasan International B.V. Holland, Woerden, The Netherlands) at a dose of 80 mg/kg and xylazine hydrochloride (Alfazyne, Alfasan International B.V. Holland) at a dose of 10 mg/kg. Electrophysiological recordings, namely electromyography (EMG) studies, were conducted on all groups upon completion of the study. EMG data sets were collected from the right and left sciatic nerves. The nerves were subjected to supramaximal stimulation at an intensity of 10 V, utilizing a pulse duration of 0.05 ms and a frequency of 1 Hz. The EMG signals were captured within a frequency spectrum from 0.5 to 5000 Hz, utilizing a sampling rate of 40 kHz per second, as demonstrated in Figure 2. The stimulation procedure involved the utilization of a bipolar subcutaneous needle electrode manufactured explicitly by BIOPAC Systems, Inc., located in Santa Barbara, CA, USA. This electrode was strategically positioned at the sciatic notch. The study employed unipolar platinum electrodes to capture compound muscle action potentials (CMAPs) originating from two to three interosseous muscles. The data analysis was conducted using the Biopac Student Lab Pro version 3.6.7 software, which BIOPAC Systems, Inc. (Goleta, CA, USA), developed. The evaluation parameters encompassed the distal latency and amplitude of the CMAP. While conducting EMG recordings, the rectal temperatures of the rats were assessed by employing a rectal probe (H.P. Viridia 24-C; Hewlett-Packard Company, Palo Alto, CA, USA). The temperature of each rat was regulated within the range of 36–37 °C through a heating pad. The trials were conducted between 10:00 a.m. and 2:00 p.m. 

### 2.4. Assessment of Motor Function via Inclined Plane Test

The motor performances of the rats were assessed using the inclined-plate test. In this experiment, the rat was positioned at an angle relative to the elongated axis of an inclined plate. The original angle of inclination of the plate was 10 degrees. The inclination angle gradually escalated, and the motor score was determined as the greatest angle of the plate on which the rat maintained its place for 5 s without experiencing a fall. The angle of the inclined plate was measured three times in each rat to determine an average value.

### 2.5. Histology and Quantitative Histochemistry 

The rats were subjected to intracardiac perfusion using a 4% formaldehyde solution to facilitate histological and quantitative immunohistochemical assessments. The distal segments, which were situated 10 mm from the transected site of the sciatic nerves, were surgically removed. Embedded in paraffin, the sciatic nerves underwent sectioning into 5 µm slices using a microtome (Leica RM 2145, Nussloch, Germany). Subsequently, these sections were subjected to staining with hematoxylin and eosin (H&E).

The number of cells was quantified in at least five randomly chosen regions to ascertain the extent of fibrosis. The calculation of the percent fibrosis score involved the division of the total cell count in the specified location by the count of enumerated cells. 

Before conducting the immunohistochemistry analysis, it was imperative to deactivate the endogenous peroxidase activity of the samples by treating them with a 10% hydrogen peroxide solution for 30 min. Following this step, the samples were blocked with normal goat serum (Invitrogen, Waltham, MA, USA) at room temperature for 1 h. Following this, the sections underwent incubation at a temperature of 4 °C for 24 h using a specific primary antibody (Santacruz Biotechnology, Dallas, TX, USA; diluted at a 1:100) that targeted nerve growth factor (NGF). 

Antibodies were detected using the rabbit immunoglobulin G-specific Histostain-Plus Bulk kit (Invitrogen), and the result was evaluated using 3,3′-diaminobenzidine (DAB). The slices were washed using phosphate-buffered saline and subsequently seen using an Olympus BX51 microscope. The digital images were captured with an Olympus C-5050 camera. Quantitative immunohistochemistry was performed on all groups and six slices from each animal. The immune-positive Schwann cells and axons were quantified by two blinded investigators employing a light microscope.

The following factors were quantified: the number of axons and the diameter of axons. The cross-sections were acquired for these specific parameters by utilizing a digital counter with the assistance of a grid. This process was conducted in six randomly selected fields: one central and five peripheral fields. The magnification used for this procedure was 20×.

### 2.6. Nerve Biochemical Analysis 

Following the sacrifice, both sciatic nerves were promptly excised and preserved at a temperature of −20 °C until further biochemical examination. The distal segments 10 mm away from the transected location of the sciatic nerves were subjected to homogenization using a glass homogenizer in a volume of 5 times the amount of phosphate-buffered saline (pH 7.4). The resulting mixture was then centrifuged at a speed of 5000× *g* for 15 min. Subsequently, the liquid portion (supernatant) was gathered, and the overall protein concentration in the mixtures was assessed utilizing Bradford’s technique, employing bovine serum albumin as a reference standard [26].

Galectin-3, nerve growth factor (NGF), and GDF-11 concentrations were quantified in the supernatants using commercially available enzyme-linked immunosorbent assay (ELISA) kits designed for rats. The measurements of the samples from each animal were conducted twice, adhering to the parameters specified by the manufacturer. The measurement of absorbances was performed using a microplate reader (MultiscanGo, Thermo Fisher Scientific Laboratory Equipment, Portsmouth, NH, USA).

### 2.7. Statistical Analysis 

The statistical analysis was performed utilizing IBM SPSS version 20. The data are displayed using each group’s mean value and standard deviation (SD). The statistical methodology utilized to conduct multiple comparisons in this study was the one-way analysis of variance (ANOVA). A probability value that is less than 0.05 (*p* < 0.05) was considered to have statistical significance. The data were reported as the mean value plus or minus the standard error of the mean (SEM). 

## 3. Results

**Electrophysiological Assessments:**

The nerve function assessment in the various groups was conducted by using EMG. The findings are depicted in Figure 2 and Table 1.

EMG CMAP Latency (ms): In the control group, the latency was 2.25 ± 0.14 ms. The placebo group significantly increased to 3.63 ± 0.11 ms (*p* < 0.05, different from the control). However, in the experimental group, the latency was 3.42 ± 0.15 ms, showing improvement compared to the placebo group (Table 1).

EMG CMAP Amplitude (mV): In the control group, the amplitude was 13.3 ± 1.5 mV. The placebo group exhibited a significant reduction in amplitude (2.03 ± 0.2 mV, *p* < 0.05, different from the control). In contrast, the experimental group improved in amplitude (8.3 ± 0.4 mV, *p* < 0.05, different from the control).

Inclined Plane Score (°): The control group had a motor score of 82.6 ± 2.9 degrees. In the placebo group, the motor score significantly decreased to 28.7 ± 6.4 degrees (*p* < 0.01, different from the control). However, in the experimental group, the motor score improved to 77.1 ± 5.5 degrees (## *p* < 0.01, different from the placebo group) (Table 1).

**Histological and Immunohistochemical Analysis:**

**Figure 3:**
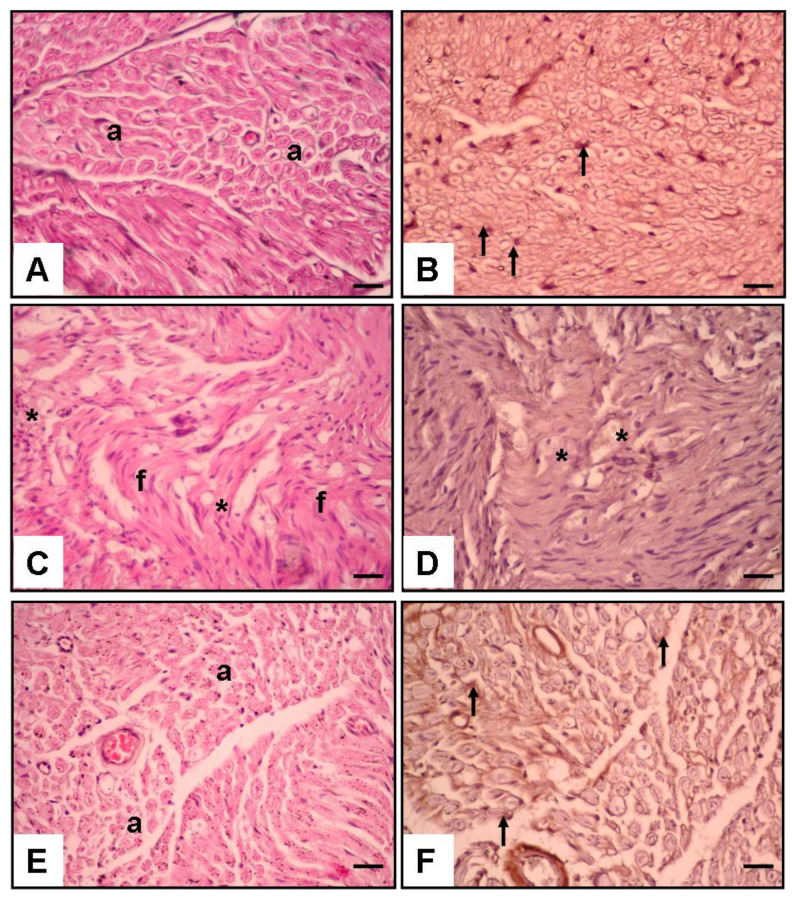
Histological results of different groups at 20× magnification with hematoxylin and eosin and NGF immunostaining. (**A**,**B**): Control group. Normal axon (a) and Schwann cell (arrow). (**C**,**D**): Surgery and saline group. Increased fibrosis (f), very diminished axon, Schwann cell, and NGF immunoexpression (asterisk). (**E**,**F**): Surgery and liraglutide group. Increased axon (a), Schwann cell, and NGF immunoexpression (arrow).

Histological and immunohistochemical assessments were performed to evaluate axon morphology and NGF immunoexpression. The results are shown in Figure 3 and Table 2.

**Histological Assessment (Figure 3):**

Figure 3 provides a detailed histological examination of nerve tissue at 20× magnification, utilizing hematoxylin and eosin staining and NGF immunostaining to assess structural integrity and neurotrophic factor expression.

*Figure 3A,B: Control Group:*

Microscopic examination of the control group revealed a histologically intact nerve structure characterized by normal axon morphology (a) and well-defined Schwann cells (arrow). This baseline condition was a reference for subsequent comparisons (Figure 3).

*Figure 3C,D: Surgery and Saline Group:*

In contrast, the histological sections from the placebo group depicted noticeable abnormalities, including increased fibrosis (f) and a significant reduction in the axon, Schwann cell density, and NGF immunoexpression (asterisk). These observations suggest a compromised regenerative response without treatment (Figure 3).

*Figure 3E,F: Surgery and Liraglutide Group:*

Histological analysis of the experimental group showcased distinct improvements compared to the placebo group. Increased axon density (a), well-preserved Schwann cells, and enhanced NGF immunoexpression (arrow) were evident, indicating a more favorable regenerative environment in response to liraglutide treatment (Figure 3).

NGF Immunoexpression on Schwann Cells (%): In the control group, the NGF immunoexpression on Schwann cells was 91.4 ± 8.3%. The placebo group exhibited significantly reduced NGF immunoexpression (5.2 ± 0.6%, *p* < 0.05, different from the control). In contrast, the experimental group exhibited improved NGF expression (73.6 ± 4.05%, ## *p* < 0.01, different from the placebo group) (Table 2).

Total Axon Number: The total axon number in the control group was 289.1 ± 16.3. The placebo group exhibited a significantly reduced total axon number (17.6 ± 2.5, *p* < 0.01, different from the control). In contrast, the experimental group exhibited an improved total axon number (103.2 ± 9.2, ## *p* < 0.01, different from the placebo group) (Table 2).

Axon Diameter (µm): The axon diameter in the control group was 3.46 ± 0.15 µm. The placebo group exhibited a significant reduction in axon diameter (1.83 ± 0.24 µm, *p* < 0.05, different from the control). In contrast, the experimental group exhibited an improved axon diameter (3.12 ± 0.17 µm, # *p* < 0.05, different from the placebo group) (Table 2).

Fibrosis Score (%): The fibrosis score in the control group was 0.8 ± 0.1%. The placebo group exhibited a significant increase in the fibrosis score (84.6 ± 7.5%, *p* < 0.01, different from control). In contrast, the experimental group exhibited a reduced fibrosis score (7.5 ± 2.6%, ## *p* < 0.01, different from the placebo group) (Table 2).

**Nerve Biochemical Analysis:
**

Biochemical analyses were conducted to assess various factors in the nerves. The results are shown in Table 3.

MDA (Malondialdehyde) Level (nmol/μg): In the control group, the MDA level was 103.1 ± 9.5 nmol/μg. The placebo group exhibited a significant increase in the MDA level (198.2 ± 10.6 nmol/μg, *p* < 0.05, different from the control). However, the experimental group exhibited a reduced MDA level (121.5 ± 8.1 nmol/μg, # *p* < 0.05, different from the placebo group) (Table 3).

Nerve Galectin-3 Level (pg/mg): In the control group, the galectin-3 level was 6.3 ± 0.1 pg/mg. The placebo group exhibited a significant increase in galectin-3 level (22.6 ± 0.9 pg/mg, *p* < 0.05, different from control). However, the experimental group showed a reduction in the galectin-3 level (10.4 ± 0.6 pg/mg, # *p* < 0.05, different from the placebo group) (Table 3).

Nerve NGF Level (pg/mg): In the control group, the NGF level was 26.7 ± 0.5 pg/mg. The placebo group exhibited a significant reduction in the NGF level (10.1 ± 2.3 pg/mg, *p* < 0.05, different from the control). However, the experimental group exhibited an improved NGF level (21.2 ± 1.1 pg/mg, # *p* < 0.05, different from the placebo group) (Table 3).

Nerve GDF-11 Level (pg/mg): In the control group, the GDF-11 level was 17.8 ± 1.09 pg/mg. The placebo group exhibited a significant reduction in the GDF-11 level (9.9 ± 0.5 pg/mg, *p* < 0.05, different from control). However, the experimental group exhibited a slightly reduced GDF-11 level (13.1 ± 1.6 pg/mg, *p* < 0.05, different from the placebo group) (Table 3).

## 4. Discussion

The restorative results of peripheral nerve damage through spontaneous regeneration in nerve injury are often suboptimal even following microsurgical intervention, especially in cases of severe injury, compelling drug studies to continue to reduce the negative consequences of this situation [27]. The study’s results demonstrate a multifaceted positive impact of liraglutide on peripheral nerve injury. The combination of improved electrophysiological parameters, axon morphology, reduced fibrosis, decreased oxidative stress, and increased neurotrophic factor expression presents a strong argument for the potential clinical utility of utilizing liraglutide for managing peripheral nerve injuries. 

There are experimental and clinical studies on liraglutide in which neuroprotection has been shown. However, it still needs to be clarified in which way liraglutide provides this protection. Antioxidant, anti-apoptotic, and anti-inflammatory properties have not been fully described [28]

Trauma-induced peripheral nerve injury is a significant contributor to global disability. Following a crush injury, an initial period of injury is followed by a more prolonged secondary injury period characterized by oxidative stress, inflammation, edema, and apoptosis [29].

In this study, we observe a decrease in MDA, which results from liraglutide’s antioxidant properties. Multiple studies declare that liraglutide’s activation of GLP-1 receptors significantly decreased nitro-oxidative stress with endotoxemia, as reported in a study [30]. Liraglutide was found to reduce the oxidative stress and fatty degeneration induced by oxidized LDL through the regulation of AMPK signaling, as demonstrated in a study [28]. A study by Sato et al. [28] described reduced reactive oxygen metabolites in peripheral blood in liraglutide-treated rats. They also found that liraglutide reduced oxidative stress, as in this study. In this study, the main therapeutic effect may be considered to be through suppression of oxidative stress.

On the other hand, in a study conducted by Chen et al., they emphasize that excessive oxidative stress has been found to impair axons’ functionality by hastening microtubule degradation [29]. In this study, admission of the liraglutide provoc the augmentation of total axon number and diameter. For evaluating locomotor recovery, inclined plane test scores were used at the end of 12 weeks. Similar results were found in the study of Chen et al., which studied liraglutide in spinal cord injury, and he also found that inclined plane test results improve with liraglutide admission [29]. The results of this study indicate that liraglutide may augment functional recuperation after peripheral nerve injury.

The electrophysiological parameters were assessed using EMG to examine the nerve regeneration process. Electrophysiological tests are frequently employed as the primary assessment modality in experimental models of peripheral nerve damage [30]. The CMAP is a quantitative measure that provides insights into the characteristics of sensory-motor fibers and the magnitude of the innervated motor units [31,32]. Our study’s findings show no marginal decrease in CMAP latency in the surgery+liraglutide group compared to the surgery+saline group. However, there was a significant increase in CMAP amplitude compared to the aforementioned groups. This shows that the nerve latency needs more than 12 weeks of treatment for liraglutide. 

In contrast, Yamamoto et al. [33] conducted a study wherein they administered exendin-4 treatment for 14 days in cases of peripheral nerve injury. The researchers observed that repeated intraperitoneal administration of exendin-4 resulted in significant improvements in motor function recovery, electrophysiological data, and histological findings in rats with sciatic nerve crush injuries.

Given the observed increase in GLP-1R immunoreactivity in Schwann cells following administration of exendin-4 [12], it is plausible to suggest that exendin-4 facilitates axonal regeneration by stimulating Schwann cells via GLP-1R activation. Similar to these findings in our study, the percentage of NGF immunoexpression in Schwann cells in the liraglutide-treated group was significantly increased. Moreover, in a study that examined the long-term effects of exendin-4 administration (over 12 weeks) [5], rats underwent sciatic nerve transection and received daily injections of 10 μg of Ex-4. Similarly, in the GLP-1 analog Ex-4, there was increased hindlimb muscle strength, as shown by latency and amplitude improvements.

Furthermore, the histomorphometric analysis revealed that liraglutide administration significantly increased NGF immunoexpression, total axon number, and diameter, reducing fibrosis scores in the injured nerves. NGF is crucial in promoting axonal growth and nerve regeneration [34]. The results are consistent with studies highlighting NGF as a potential therapeutic target to enhance nerve regeneration [35,36]. Restoring NGF levels and axonal parameters through liraglutide administration substantiates its potential as a therapeutic intervention for peripheral nerve injuries.

Galectin-3 is a molecule that controls many processes, ranging from short-term inflammation to long-term inflammation and tissue fibrogenesis [37]. Gustavsson et al. [38] conducted a study that showed galectin-3 can stop Schwann cells from multiplying in sciatic nerve segments grown in a lab. Many people know that myelin clearance, Schwann cell proliferation, and the success of PNS regeneration are all connected. In a study by Narciso [39], mice that did not have the galectin-3 gene healed and grew back better than mice that did have this gene. This was likely because they had more Schwann cells and β-catenin expression. Our study found that galactin-3 levels went up in the nerve injury group but down in the surgery+liraglutide group. If this is true, liraglutide stops nerves from healing themselves.

Due to the anti-inflammatory effects of GDF-11, it has been studied exogenously in experimental studies in processes such as fatty liver, renal injury, spinal cord injury, peripheral nerve injury, and cerebral hemorrhage, and positive results have been found regarding its protective and healing effects [40,41,42]. Previous research has demonstrated that GDF-11 can impede cell apoptosis [43], facilitate the growth of axons and synapses [44,45], and initiate neuronal regeneration. In our injury model, GDF-11 decreased in the saline group and increased in the liraglutide group. In this way, liraglutide suppresses the apoptotic process and supports the regeneration of the injured nerve. Studies on the exogenous use of GDF-11 in peripheral nerve injury should be increased.

Liraglutide improved nerve recovery functionally and histologically. These results suggest that liraglutide may promote nerve regeneration and repair. Moustafa et al. proved that liraglutide showed histopathologically improved STZ-induced peripheral neuropathy with anti-glycemic, antioxidant, and anti-inflammatory effects [46]. Similarly, Liu et al. [12] found that exendin-4 can enhance axonal regeneration and promote the formation of myelinated nerve fiber cells. Thus, the preservation of the distinctive histopathological integrity of the sciatic nerve is achieved in diabetic rats.

In addition to glycemic control, which is the primary use of liraglutide on the market, we also observed that liraglutide, by its nature, acts constructively in acute as well as chronic inflammatory processes, especially in peripheral nerve injury.

This study demonstrates that liraglutide administration following peripheral nerve injury mitigates the adverse effects on nerve regeneration and promotes neuroprotection. This effect might be attributed to liraglutide involvement in modulating glucose, GDF-11, and galactin-3, leading to enhanced antioxidant defense, neurotrophic support, and reduced inflammation.

## 5. Limitations

This study was conducted using an animal model; additional research is required to ascertain the efficacy and safety of liraglutide in humans. Additionally, the precise molecular mechanisms underlying the effects of liraglutide require further investigation. On the other hand, different doses can be tried in this direction using liraglutide. The long-term effects of liraglutide on rats can be assessed. Prospective randomized studies can be conducted on the EMG outcomes.

## 6. Conclusions

This study suggests that liraglutide may help patients regenerate and regain function in peripheral nerve injury. Since this study is a preclinical, experimental study, more experimental and clinical studies are needed to use GLP-1 in nerve damage.

## Figures and Tables

**Table 1:** Measurements related to surgery with liraglutide were taken in different groups. Data are expressed as the mean ± standard error of the mean (SEM). * *p*< 0.05 different from the control group, ** *p* < 0.001 different from the control group, # *p* < 0.01 different from the placebo group, ## *p* < 0.001 different from the placebo group.

	Control	Surgery and Saline (Placebo)	Surgery and Liraglutide Group (Experimental Group)
EMG CMAP latency (ms)	2.25 ± 0.14	3.63 ± 0.11 *	3.42 ± 0.15
EMG CMAP amplitude (mV)	13.3 ± 1.5	2.03 ± 0.2 **	8.3 ± 0.4 #
Inclaned plane score (°)	82.6 ± 2.9	28.7 ± 6.4 **	77.1 ± 5.5 ##

**Table 2:** Data are expressed as the mean ± SEM. * *p*< 0.05 different from the control group, ** *p* < 0.001 different from the control group, # *p* < 0.01 different from the placebo group, ## *p* < 0.001 different from the placebo group.

	Control	Surgery and Saline (Placebo)	Surgery and Liraglutide (Experimental)
NGF immunoexpression on Schwann cell (%)	91.4 ± 8.3	5.2 ± 0.6 **	73.6 ± 4.05 ##
Total axon number	289.1 ± 16.3	17.6 ± 2.5 **	103.2 ± 9.2 ##
Axon diameter, µm	3.46 ± 0.15	1.83 ± 0.24 *	3.12 ± 0.17 #
Fibrosis score (%)	0.8 ± 0.1	84.6 ± 7.5 **	7.5 ± 2.6 ##

**Table 3:** Measurements related to nerve injury and treatments with liraglutide were taken in different experimental groups. Data are expressed as the mean ± SEM. * *p*< 0.05 different from the control group, ** *p* < 0.001 different from the control group, # *p* < 0.05 different from the placebo group.

	Control	Surgery and Saline (Placebo)	Surgery and Liraglutide Group (Experimental Group)
MDA (nmol/μg)	103.1 ± 9.5	198.2 ± 10.6 *	121.5 ± 8.1 #
Nerve Galectin-3 Level (pg/mg)	6.3 ± 0.1	22.6 ± 0.9 **	10.4 ± 0.6 #
Nerve NGF Level (pg/mg)	26.7 ± 0.5	10.1 ± 2.3 *	21.2 ± 1.1 #
Nerve GDF-11 Level (pg/mg)	17.8 ± 1.09	9.9 ± 0.5 *	13.1 ± 1.6 #

## Data Availability

All the data for this study are presented in the published article. Any further details are available from the corresponding author (Ejder Saylav Bora, saylavbora@hotmail.com) upon a reasonable request.
